# Cytogenetics and Cytogenomics in Clinical Diagnostics: Genome Architecture, Structural Variants, and Translational Applications

**DOI:** 10.3390/genes16070780

**Published:** 2025-06-30

**Authors:** Concetta Federico, Desiree Brancato, Francesca Bruno, Elvira Coniglio, Valentina Sturiale, Salvatore Saccone

**Affiliations:** 1 Department of Biological, Geological and Environmental Sciences, University of Catania, 95124 Catania, Italy; federico@unict.it (C.F.); desiree.brancato@phd.unict.it (D.B.); elvira.coniglio@phd.unict.it (E.C.); valentina.sturiale@phd.unict.it (V.S.); 2 Department of Medicine and Surgery, Kore University of Enna, 94100 Enna, Italy; francesca.bruno@unikore.it

**Keywords:** nuclear genome architecture, chromosomal rearrangements, 3D genome organization, cytogenomic diagnostics, replication timing, structural variants, cancer genomics, high-resolution genomic technologies

## Abstract

The spatial organization of the genome within the nucleus is a fundamental regulator of gene expression, genome stability, and cell identity. This review addresses the central question of how nuclear genome architecture contributes to disease mechanisms and diagnostics, and how technological advances enable its clinical exploration. We first outline the principles of nuclear genome architecture, including chromosome territories, replication timing, and 3D domains, and their role in gene regulation and disease. We then explore the mechanisms and consequences of chromosomal rearrangements, and how replication dynamics intersect with epigenetic regulation and genome stability. Diagnostic tools are presented in chronological progression, from conventional cytogenetics to high-resolution genomic and single-cell techniques. A dedicated section focuses on cancer cytogenomics and its clinical implications. We further highlight emerging technologies for 3D genome and epigenome profiling and their integration into diagnostic workflows. Finally, we discuss current challenges, such as standardization and cost, and the transformative potential of multi-omics and artificial intelligence for future precision diagnostics. Overall, we provide a comprehensive overview of how cytogenetics and cytogenomics contribute to the understanding and clinical diagnosis of genetic and neoplastic diseases.

## 1. Introduction

In recent decades, genetic diagnostics have evolved from classical cytogenetics to cytogenomics, integrating chromosomal morphology with high-resolution genomic technologies. While traditional cytogenetics have long been central to identifying genomic syndromes and cancer-associated rearrangements, cytogenomics have broadened diagnostic capabilities by enabling the detection of a wider range of structural variants (SVs), including balanced and unbalanced rearrangements, inversions, duplications, and complex events such as chromothripsis [1,2,3,4].

Concurrently, the growing interest in the spatial and temporal organization of the genome has illuminated the functional relevance of three-dimensional (3D) nuclear architecture in gene regulation. Chromosome positioning, long-range chromatin interactions, and hierarchical compartmentalization into TADs, loops, and A/B compartments are functionally involved in transcriptional control and human disease [5,6,7,8]. Importantly, alterations in 3D genome topology have been implicated in diverse human pathologies, including developmental syndromes, neurogenetic disorders, and cancers, thus linking cytogenomic structural changes to functional consequences.

Within this framework, modern cytogenetics and cytogenomics have emerged as indispensable disciplines for elucidating the molecular mechanisms underlying rare genetic diseases and complex cancers. These fields now integrate high-resolution structural analysis with functional insights into genome architecture, offering progressively sophisticated diagnostic tools. This review aims to provide a comprehensive overview of cytogenomic approaches, emphasizing the need for their standardization in both research and clinical diagnostics. We focus on how alterations in nuclear genome organization and chromosomal structure contribute to disease pathogenesis and discuss current and emerging diagnostic strategies driven by technological innovation. Emphasis is placed on the integration of genome topology and single-cell technologies, which are paving the way for a new era of precision diagnostics and personalized medicine.

In this review, we address the following key questions: (i) How does the spatial organization of the genome contribute to gene regulation and disease mechanisms? (ii) What structural and topological alterations are observed in genetic and cancer-related disorders? (iii) How can cytogenetic and cytogenomic technologies be applied to improve diagnosis and prognosis? To answer these questions, this article is structured to progress from the basic principles of nuclear genome architecture (Section 2) to specific structural rearrangements (Section 3), functional layers such as replication timing and banding (Section 4), and the evolution of diagnostic technologies (Section 5). We then focus on cancer applications (Section 6) and conclude with emerging strategies based on 3D genome profiling and multi-omics integration (Section 7 and Section 8).

## 2. The Nuclear Genome Architecture in Health and Disease

### 2.1. Spatial Organization of the Genome and Chromosome Territories

The spatial organization of the genome within the interphase nucleus is a fundamental aspect of gene regulation and genomic stability. Rather than being randomly distributed, chromosomes occupy discrete chromosome territories that function as both structural domains and regulatory hubs [9,10,11,12].

These territories are not passive structural entities but are intimately involved in modulating transcriptional activity, DNA replication timing, and the DNA damage response. For instance, the radial positioning of chromosome 7 in human lymphocyte nuclei is strongly correlated with chromosomal band gene density and replication timing. Gene-rich regions tend to be localized toward the nuclear interior and replicate early during the S-phase, whereas gene-poor, heterochromatic regions are typically peripherally located and replicate later. This spatial–functional relationship underscores how nuclear architecture is tightly linked to the functional state of the genome [13,14].

To provide a plausible structural and biophysical mechanism underlying this spatial genome organization at the molecular level, the multilayer chromatin model proposed by Daban has been introduced. This model suggests that weak, repetitive interactions between nucleosomes can drive the self-assembly of chromatin into highly ordered, multilaminar structures. These configurations mirror the patterns observed in cytogenetic banding and are consistent with the functional compartmentalization within interphase nuclei. Moreover, they offer a physical substrate for the differential accessibility of genomic regions, enabling selective transcriptional regulation across distinct chromosome territories. In doing so, the model contributes both to structural integrity and to resistance against topological stress [15].

**Figure 1:**
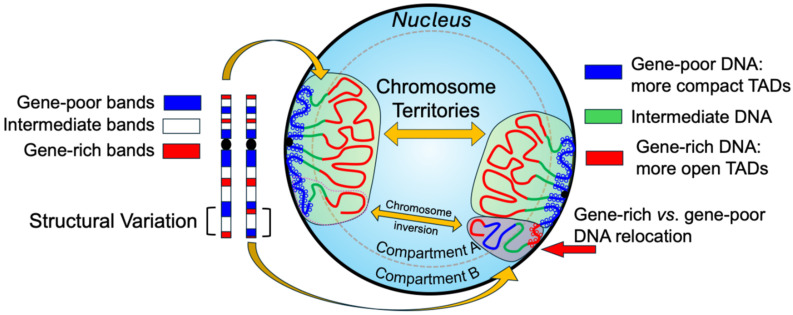
Structural variation and chromosome territory reorganization. Structural variations involving chromosomal bands with different compositional properties can lead to the repositioning of specific genes into nuclear environments with distinct regulatory characteristics. This relocation may result in ectopic expression or repression of the translocated genes. Conversely, when the involved chromosomal regions share similar compositional properties, the structural variation may be functionally neutral or asymptomatic.

Moreover, alterations in chromosome positioning and territory organization have been implicated in pathological states, including cancer and developmental disorders. Disruptions in these spatial patterns—whether through structural rearrangements or epigenetic deregulation—can lead to aberrant gene expression, enhancer hijacking, or inappropriate long-range chromatin contacts (Figure 1). As such, spatial genome organization is emerging not only as a fundamental principle of nuclear biology but also as a clinically relevant dimension of genomic medicine [5].

This concept of genome architecture as a determinant of both structural stability and functional output is further expanded by the Genome Architecture Theory (GAT), which posits that the genome operates as a hierarchical system where the spatial configuration of the karyotype encodes regulatory instructions. According to this view, karyotype coding serves as a higher-order mechanism for coordinating gene networks, maintaining genomic integrity, and defining cellular identity. By integrating this theory with structural models such as Daban’s, one obtains a multiscale view of genome function—linking nanoscale chromatin organization to global nuclear topology and disease etiology informing new diagnostic strategies that move beyond linear, gene-centric approaches. Recent studies have supported this concept by framing karyotype organization as a form of ‘system inheritance’ central to both evolution and disease [16,17,18].

### 2.2. Topologically Associating Domains and 3D Gene Regulation

**Figure 2:**
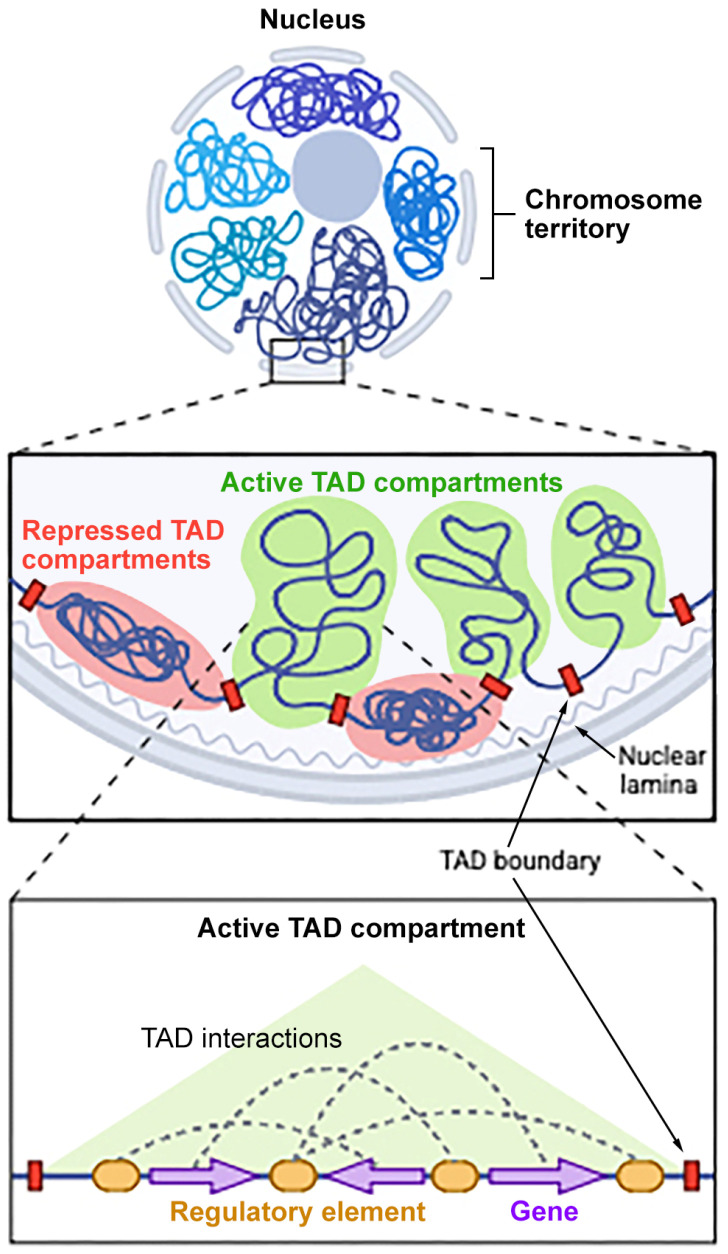
Chromosome territories and TAD compartmentalization. This schematic representation illustrates nuclear genome organization across hierarchical levels. The top panel depicts chromosome territories within the interphase nucleus. The middle panel shows the genome partitioned into Topologically Associating Domains (TADs), which are grouped into active (green) and repressed (red) compartments relative to the nuclear lamina. TADs are insulated by TAD boundaries, which maintain regulatory fidelity. The bottom left panel zooms into an active TAD compartment, highlighting intra-TAD chromatin interactions between regulatory elements (e.g., enhancers) and target genes via chromatin loops, which enable precise gene control.

Within chromosome territories, the genome is further organized into topologically associating domains (TADs), which are self-interacting genomic regions that enable the coordinated activity of genes and regulatory elements within well-defined boundaries [19,20]. TADs serve as fundamental units of 3D genome organization, insulating gene regulatory interactions and preventing promiscuous enhancer–promoter contacts across domain boundaries (Figure 2).

The structural integrity of TADs is maintained by chromatin loops anchored by architectural proteins such as CTCF (CCCTC-binding factor) and the cohesin complex, which facilitate long-range interactions between promoters and distant enhancers—crucial for precise and dynamic transcriptional regulation [21].

These loops are not only essential for the proper spatial configuration of the genome but also underpin cell type-specific transcriptional programs by enabling or restricting physical contact between regulatory elements. The disruption of these looping structures has been associated with pathological gene misexpression, as seen in various developmental disorders and malignancies.

This 3D genome architecture is closely linked to the cell cycle: during the interphase, chromatin adopts an open and flexible conformation, allowing dynamic chromatin remodeling and transcriptional activity. In contrast, as cells enter mitosis, chromatin undergoes extensive condensation into highly compacted metaphase chromosomes. Despite this structural transition, the linear order of chromatin loops is preserved, which is believed to facilitate the faithful re-establishment of interphase nuclear organization after cell division into daughter cells [22]. This cyclical modulation of chromatin topology highlights the plasticity of nuclear architecture and its central role in maintaining cellular identity and genomic stability across generations.

Beyond TADs, it is important to contextualize genome organization within the broader framework of chromosome structure and nuclear architecture. Classical cytogenetic methods such as FISH banding continue to provide valuable insights into large-scale chromosomal organization and complement high-resolution genomic approaches. Notably, Cremer has emphasized the significance of integrating cytogenetic banding patterns with three-dimensional nuclear organization to better understand chromosomal behavior and function during the interphase [23]. This integrative perspective highlights how large-scale chromosome territories and their banding characteristics provide a scaffold within which finer-scale structures like TADs operate, thereby enriching our understanding of genome regulation in both physiological and pathological contexts.

### 2.3. Nuclear Architecture Alterations in Genetic and Neoplastic Disorders

Spatial alterations in nuclear architecture have been documented in several genetic and neoplastic disorders, highlighting the functional relevance of genome topology in human disease. For instance, in pediatric acute myeloid leukaemias (AML) carrying the recurrent t(7;12)(q36;p13) translocation, the *MNX1* gene (formerly *HLXB9*) is repositioned within the nucleus and aberrantly expressed, demonstrating how changes in 3D genomic localization—not just gene disruption or fusion—can misregulate transcription by disrupting the regulatory element [5,24]. Beyond specific gene effects, the intrinsic genomic features of chromosomal bands, such as gene density and GC content, are associated with the phenotypic consequences of chromosomal rearrangements. Certain rearrangements disrupt TADs, rewire enhancer–promoter interactions, or reposition chromatin within the nuclear space, ultimately leading to pathological outcomes including congenital syndromes or oncogenic transformation. Conversely, some rearrangements affect regions with low regulatory complexity and maintain the spatial organization of the genome, persisting as chromosomal polymorphisms in the general population, being transmitted without pathological outcomes. This distinction underscores the critical role of nuclear genome organization in mediating genomic plasticity and evolutionary processes [5,25]. When nuclear organization is disrupted—such as through the mislocalization of genes, the separation of enhancers from their target genes, or the relocation of chromatin domains between transcriptionally active and repressive compartments—dysregulated gene expression may ensue. Conversely, rearrangements that preserve the integrity of nuclear topology are less likely to result in disease phenotypes, even when they involve considerable genomic material [26]. Thus, the interplay between genome structure and function offers a mechanistic explanation for the variable penetrance and expressivity observed in individuals with similar cytogenetic abnormalities. Moreover, it positions nuclear architecture as a key modulator of genomic plasticity, with implications for both evolutionary biology and clinical cytogenomics.

### 2.4. Diagnostic Implications and Future Perspectives of Functional Cytogenomics

Broader dysfunctions in nuclear architecture have been documented in cancer and genetic disorders. In cancer, alterations in chromosome territory positioning, TAD integrity, and chromatin loops can activate oncogenes and suppress tumor suppressors, promoting hallmark features such as uncontrolled proliferation and genomic instability [27]. Similarly, in disorders like laminopathies or Rett syndrome, mutations in nuclear structural proteins or chromatin remodelers cause nuclear disorganization, impairing epigenetic regulation and increasing susceptibility to DNA damage [28,29,30,31,32,33]. Beyond structural rearrangements, even single nucleotide polymorphisms (SNPs) located within non-coding regulatory regions can impact nuclear architecture with critical sequences involved in TAD organization which may affect the activation or silencing of specific genes. For instance, common variation in the regulatory architecture of the *OCA2* gene locus affects pigmentation traits such as human eye color, illustrating how non-coding variants can influence phenotype through spatial genomic mechanisms [34,35].

Understanding these spatial alterations has driven the development of diagnostic strategies that integrate structural, morphological, and dynamic genome features. Notably, Misteli introduced the concept of the self-organizing genome, suggesting that 3D genome organization is not just a functional outcome but an active driver of gene regulation and genomic stability [36]. This view has catalyzed technological advances in genome visualization. Among them, optical genome mapping detects large-scale structural variants, while super-resolution microscopy enables the nanoscale visualization of chromatin domains and nuclear compartments. Recently, Deng et al. [37] emphasized that alterations in 3D chromatin architecture are tightly linked to transcriptional control in cancer, underscoring the diagnostic and prognostic potential of spatial genome analysis. These insights support the clinical implementation of nuclear architecture profiling as a powerful complement to sequence-based diagnostics, paving the way for a more comprehensive understanding of disease mechanisms and personalized therapeutic strategies.

## 3. Chromosomal Rearrangements: Mechanisms and Functional Implications

### 3.1. Mechanisms of Formation

**Figure 3:**
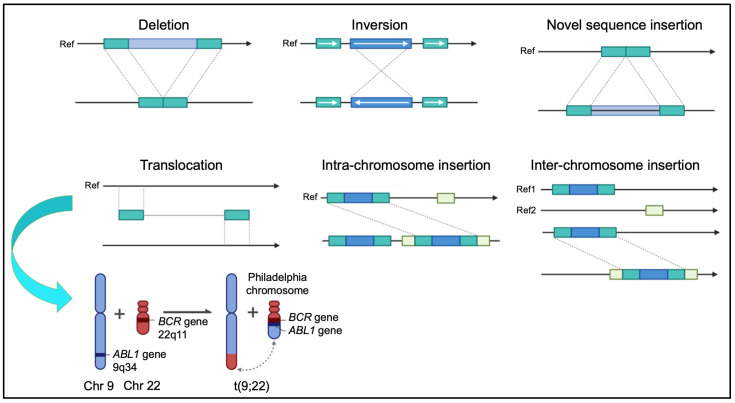
Overview of genome structural variations, including deletions, inversions, insertions, and translocations. These rearrangements can disrupt gene function, create fusion genes, or alter gene regulation, and are often associated with developmental disorders and cancer. As a representative example, the Philadelphia chromosome translocation t(9;22)(q34;q11) is shown, resulting in the *BCR::ABL1* fusion gene implicated in chronic myeloid leukemia. This example is provided to illustrate how structural genome alterations, including gene fusions, can play a central role in disease pathogenesis—among many other chromosomal rearrangements that impact human health.

Structural variations (SVs), including translocations, inversions, deletions, and duplications, contribute significantly to genomic diversity and are closely associated with numerous genetic disorders and cancers (Figure 3). These rearrangements can exert their effects through diverse mechanisms: by disrupting gene function, altering regulatory elements, or modifying the 3D genome architecture. In particular, SVs may reposition enhancers, insulators, or architectural proteins, thereby altering TAD boundaries and reshaping local and long-range gene regulatory interactions [7,38].

The formation of structural variants is driven by multiple DNA damage repair and replication-associated mechanisms, each characterized by distinct molecular signatures and clinical outcomes:

- **Non-Homologous End Joining (NHEJ):** Repairs double-strand breaks without a homologous template, often generating small insertions or deletions and facilitating both balanced and unbalanced translocations. This mechanism is commonly implicated in lymphoid malignancies, where antigen receptor gene rearrangements misfire [39].
- **Fork Stalling and Template Switching (FoSTeS):** Occurs during DNA replication when stalled forks switch templates, resulting in complex rearrangements such as deletions, duplications, and copy number variants [40].
- **Chromothripsis:** A catastrophic event causing chromosome fragmentation and erroneous reassembly. This process can generate tens to hundreds of clustered rearrangements and is increasingly recognized as a driver of aggressive cancers such as glioblastoma, osteosarcoma, and some hematologic malignancies [41].
- **Chromoanasynthesis:** A replication-based mechanism involving serial template switching during DNA synthesis, giving rise to highly complex SVs with duplications, triplications, and microhomology at breakpoint junctions. It is frequently associated with congenital malformation syndromes and neurodevelopmental disorders [42].
- **Chromoplexy:** A process involving coordinated, interdependent DNA strand breaks and re-ligation events across multiple chromosomes. It generates complex but often balanced rearrangements and is particularly enriched in prostate and other epithelial cancers, where it can simultaneously affect multiple oncogenes and tumor suppressors [43].

These distinct mechanisms underscore the complexity of structural variant formation and highlight how the genomic context, repair pathway activity, and cell type specificity collectively shape the mutational landscape. In particular, the interaction between SV formation and 3D genome organization introduces an additional layer of functional consequence, as rearrangements that span or reshape TADs can lead to enhancer hijacking, neo-TAD formation, or inappropriate gene activation. Table 1 summarizes the primary pathways driving these events, outlines the types of structural variants typically observed, and lists representative clinical associations.

In addition to these principal mechanisms, several less frequent but biologically relevant pathways contribute to genomic structural instability. Break-Induced Replication (BIR) is a DNA repair process that operates in response to one-ended double-strand breaks, particularly under replication stress. This mechanism can lead to non-reciprocal translocations, segmental duplications, and copy number amplifications, and has been implicated in both cancer progression and genomic disorders [44]. Non-Allelic Homologous Recombination (NAHR), mediated by low copy repeats, is a recurrent source of rearrangements seen in syndromes such as DiGeorge and Williams–Beuren [45]. Finally, retrotransposition-mediated events, including LINE-1 (or L1) insertions, can disrupt chromosomal architecture and contribute to disease-causing SVs. L1 insertions may lead to insertional mutagenesis, exon disruption, or structural rearrangements, and have been linked to both germline and somatic structural variants in human disease [46].

### 3.2. Functional Impact

Structural variants can profoundly impact genomic function through a variety of molecular mechanisms that disrupt gene structure, regulatory networks, and chromatin architecture. These alterations contribute to a wide range of developmental anomalies, congenital syndromes, and malignancies. Below, we outline the major functional consequences of SVs:

- **Gene Disruption and Regulatory Alterations:** SVs may interrupt coding sequences or reposition genes relative to regulatory elements (e.g., enhancers). Such disruptions may lead to a loss of gene function, inappropriate expression, or dosage imbalances [47]. For example, duplications upstream of the *IHH* gene cause ectopic enhancer interactions leading to digit malformations such as brachydactyly or syndactyl [48].
- **TAD Disruption:** SVs can break or fuse TAD boundaries, altering chromatin loops and long-range gene regulation. SVs such as deletions, duplications, and inversions can fuse or separate TADs, alter chromatin loop dynamics, and facilitate inappropriate enhancer–promoter contacts. This “enhancer hijacking” mechanism can lead to tissue-specific gene misexpression, underlying some developmental disorders and cancers, as seen in duplications near the *EPHA4* locus that cause limb abnormalities [49]. In cancers, TAD disruptions may activate proto-oncogenes or silence tumor suppressors through similar architectural perturbations.

Taken together, the mechanistic diversity of SVs underscores the necessity of integrating genome structural analysis with functional and spatial genomics to achieve a comprehensive understanding of disease. Modern diagnostic approaches must therefore not only detect the presence of SVs, but also interpret their regulatory consequences in the context of 3D chromatin organization and gene expression.

### 3.3. Clinically Relevant Rearrangements

Clinically relevant SVs encompass a wide spectrum with major implications in cancer biology and genetic syndromes. These rearrangements play critical roles in disease pathogenesis, prognosis, and therapeutic decision making. Below is an overview of key SV classes and their clinical significance:

- **Balanced Translocations:** Balanced chromosomal translocations often result in gene fusions with oncogenic potential. A classical example is the *BCR::ABL1* fusion in chronic myeloid leukemia (CML) [50]. Similarly, the t(15;17) translocation in acute promyelocytic leukemia (APL) produces the *PML::RARA* fusion, rendering the disease highly responsive to differentiation therapy with all-trans retinoic acid (ATRA) and arsenic trioxide [51].
- **Interstitial Deletions:** Deletions on chromosome arms, particularly on 5q and 20q, are frequent in myeloid malignancies such as myelodysplastic syndromes (MDS), often associated with a favorable prognosis by disrupting tumor suppressor genes or regulatory regions [52]. In contrast, deletions affecting 7q and 17p correlate with aggressive disease and poor outcomes [53].
- **Complex SVs and Chromothripsis:** Chromothripsis causes massive localized chromosomal rearrangements that drive oncogenesis by forming oncogenic fusions or deleting tumor suppressors. It is observed in hematologic malignancies and solid tumors like neuroblastoma and glioblastoma, which are frequently linked to treatment resistance and a poorer prognosis [54,55].
- **Constitutional Rearrangements:** Microdeletions and duplications cause syndromes such as 22q11.2 deletion syndrome (DiGeorge syndrome), affecting the cardiac, immune, and neurodevelopmental systems [52]. Similarly, microdeletions/duplications at 15q13.2–q13.3 and cryptic chromosomal aberrations contribute to autism spectrum disorder and intellectual disability [56,57]. These rearrangements are often cryptic and may be missed by conventional cytogenetic techniques.

Recent advances in long-read sequencing and optical genome mapping are revealing previously undetectable complex SVs, enhancing diagnostic precision and enabling personalized therapeutic strategies [58]. Thus, clinically relevant chromosomal rearrangements span a wide range of genomic alterations—from balanced translocations central to leukemia pathogenesis, to chromothripsis driving solid tumors, and constitutional SVs underlying developmental syndromes. Understanding these rearrangements is essential for diagnosis, prognosis, and precision medicine approaches.

## 4. Replication Timing and Chromosome Banding in Cytogenomic Diagnostics

The temporal regulation of DNA replication—known as replication timing (RT)—is a fundamental determinant of genome organization and function. During the S-phase of the cell cycle, distinct chromosomal regions replicate at specific times, which closely correlate with transcriptional activity, epigenetic modifications, and the three-dimensional (3D) architecture of the genome. Early-replicating domains are frequently located in the nuclear interior and tend to co-localize with active compartments (A compartments), whereas late-replicating regions often reside at the nuclear periphery or in lamina-associated domains (LADs), corresponding to inactive B compartments. Changes in replication timing are increasingly recognized as markers of cell identity, differentiation state, and pathological transformation. During development, RT profiles undergo tightly regulated reprogramming to support lineage-specific transcriptional programs. Conversely, aberrant replication timing is observed in various cancers and developmental disorders, where it contributes to genomic instability, epigenetic deregulation, and altered nuclear architecture [59,60,61,62].

### 4.1. Replication Timing and Nuclear Architecture

Replication timing is a highly conserved and cell type-specific feature that reflects the precise temporal order by which different genomic regions are duplicated during the S phase. Rather than being a passive consequence of DNA synthesis, RT acts as an active marker of chromatin functional state [60]. Early-replicating domains are generally gene-rich, transcriptionally active, and associated with open chromatin marks, such as accessible chromatin, a high GC content, and an enrichment in active histone marks like H3K4me3 and H3K27ac. In contrast, late-replicating regions tend to be gene-poor, transcriptionally repressed, and associated with heterochromatic features, including H3K9me3, DNA methylation, and spatial proximity to the nuclear lamina [61,63,64].

Importantly, the 3D genome organization within the nucleus strongly correlates with RT. Chromosomal domains that replicate synchronously often cluster spatially, co-localizing with transcriptional hubs or repressive compartments [65]. Notably, early-replicating domains are preferentially located in the nuclear interior, while late-replicating domains are enriched at the nuclear periphery or near the nucleolus, indicating a spatial regulation of replication timing linked to nuclear architecture [66]. Replication domains (RDs) correspond closely to topologically associating domains (TADs), with replication order highly conserved across cell types and species. This correspondence suggests that the temporal and spatial regulation of DNA replication is coordinated through 3D genome topology [60,61,63]. Architectural proteins such as Rif1 contribute to the spatial organization and timing control of these domains through nuclear anchoring mechanisms [67,68].

### 4.2. Replication Timing as a Functional Marker

RT profiles are dynamic during development and differentiation, reflecting changes in chromatin organization and gene regulation. Domains can switch their replication timing between early and late S-phase as cells transition from pluripotency to differentiated states, suggesting a role for RT in lineage specification. These RT switches are not stochastic but follow defined patterns that are reproducible and cell type-specific, suggesting that RT plays a functional role in cell fate determination and lineage specification [69]. Such replication timing reprogramming is tightly coordinated with changes in chromatin accessibility, histone modification landscapes, and the activation or silencing of lineage-specific genes. For instance, regions that gain early replication during differentiation often correspond to genes that become transcriptionally active in the new lineage, while those that shift to late replication frequently correspond to developmentally repressed loci. This temporal restructuring is therefore considered a hallmark of epigenome remodeling during development.

Moreover, RT domains coincide with TADs whose boundaries are enriched in CTCF-binding sites and active regulatory elements [63], underscoring the link between replication timing, chromatin structure, and genome regulation.

The dynamic nature of RT during development implies that it is not merely a reflection of chromatin state but may actively participate in the regulation of transcriptional programs. RT shifts may contribute to the spatial repositioning of chromatin within the nucleus—for instance, by relocating loci from the transcriptionally repressive nuclear periphery to the active nuclear interior—thereby influencing their functional state. As such, RT is emerging as a key integrator of nuclear architecture, chromatin structure, and transcriptional control.

### 4.3. Epigenetic Regulation and Genome Stability

Late-replicating regions frequently overlap with lamina-associated domains (LADs)—large heterochromatic regions tethered to the nuclear periphery—and are enriched in heterochromatic histone modifications such as H3K9me3 and H3K27me3 [70]. These domains are generally gene-poor, transcriptionally silent, and display reduced chromatin accessibility, reflecting their repressed functional state and peripheral nuclear localization. Crucially, late-replicating regions are associated with a higher incidence of genomic instability. They exhibit elevated mutation rates and structural variation susceptibility, likely due to replication stress and less efficient DNA repair in the late S-phase [71]. Moreover, because late-replicating domains are often replicated under suboptimal conditions, they may undergo incomplete or error-prone replication, contributing to DNA double-strand breaks, chromosomal rearrangements, and epigenetic dysregulation. These features make them hotspots for genomic lesions, particularly in rapidly proliferating cells, such as in embryogenesis and tumorigenesis. Thus, RT can serve as an indicator of genomic instability, with significant implications for cancer and developmental disorders. In cancer, late-replicating regions are frequently affected by somatic copy number alterations, chromothripsis, and mutational clusters, while in congenital diseases, rearrangements involving late domains may lead to the dysregulation of gene networks essential for development. Finally, RT not only reflects the chromatin state and 3D genome architecture, but also serves as a functional indicator of replication fidelity and genome integrity. Integrating RT profiling with mutation mapping, structural variation analysis, and chromatin topology offers a powerful strategy for identifying the vulnerable regions of the genome and understanding their role in disease pathogenesis.

### 4.4. Chromosomal Banding and Epigenetic Organization

Given their shared genomic determinants and functional implications, replication timing and chromosome banding are considered together here to emphasize their convergence in both structural and diagnostic contexts.

Pioneering work by Dutrillaux and colleagues, along with subsequent studies, demonstrated that early- and late-replicating genomic regions correspond to specific cytogenetic bands: early replication is typically associated with R-bands (which appear as G-light bands in GTG banding; gene-rich, GC-rich regions), while late replication corresponds to G-dark bands (gene-poor, GC-poor regions) [64,72]. Additionally, very early-replicated regions can be highlighted by T-banding (telomeric banding). These findings have since been confirmed by numerous studies showing that replication timing profiles closely mirror chromosomal banding patterns and correlate with key genomic features such as gene density and base composition.

Importantly, these characteristics also influence chromatin spatial positioning within the interphase nucleus: early-replicating, gene-rich, GC-rich regions (corresponding to R-bands) tend to localize in the nuclear interior, whereas late-replicating, gene-poor, GC-poor regions (corresponding to G-dark bands) are typically found near the nuclear periphery [73]. By presenting replication timing and GTG banding together, we aim to underline their interconnectedness and relevance to nuclear genome organization—an aspect central to this manuscript.

Table 2 summarizes the principal features of chromosomal bands in relation to their replication timing and functional attributes.

### 4.5. Replication Stress and Disease Mechanisms

Replication stress, defined as the slowing or stalling of replication forks often due to nucleotide depletion, DNA lesions, or transcription–replication conflicts, disproportionately affects late-replicating regions. These regions have fewer origins of replication and limited time to complete DNA synthesis before mitosis, making them hotspots for chromosomal fragility, rearrangements, and copy number variants (CNVs) [79]. A well-documented consequence of replication stress is the expression of common fragile sites (CFSs), such as FRA3B and FRA16D, which are prone to forming gaps or breaks under replication stress, are frequently located in late-replicating domains and are commonly altered in cancers [80]. The instability of fragile sites under replication stress reflects not only their inherent structural properties but also their suboptimal chromatin environment, which hinders timely replication and DNA repair. This vulnerability is increasingly recognized as a driver of chromosomal instability in tumorigenesis and is being explored as a potential biomarker for cancer risk and progression.

### 4.6. Chromosomal Rearrangements in Human Pathologies

Structural rearrangements including deletions, duplications, inversions, and translocations frequently localize to late-replicating regions and can cause gene disruption or dysregulation. These rearrangements are associated with diverse pathologies including neurodevelopmental disorders and malignancies, highlighting the relevance of RT in genomic disease mechanisms [78].

Replication timing also influences DNA repair pathway choice: DNA damage in the early S-phase tends to be repaired via high-fidelity homologous recombination (HR), whereas damage arising in the late S-phase is more often resolved through error-prone non-homologous end joining (NHEJ), contributing to chromosomal instability [81,82]. This replication timing-dependent shift in DNA repair fidelity helps explain why late-replicating regions are disproportionately affected in contexts of genomic instability, such as in cancer, where defective checkpoint responses exacerbate the accumulation of DNA damage. Moreover, many fragile sites—regions prone to breakage under replication stress—are embedded within late-replicating chromatin and contribute to tumor-specific structural variants and copy number alterations.

In summary, replication timing integrates genome function, nuclear structure, and stability, providing critical insights for understanding chromosomal disorders and advancing cytogenomic diagnostics.

## 5. Diagnostic Technologies: From Conventional Cytogenetics to High-Resolution Genomics

The evolution of diagnostic technologies has revolutionized the field of clinical cytogenetics and cytogenomics, significantly expanding the ability to identify and characterize chromosomal and genomic abnormalities. What once relied solely on classical techniques—such as G-banding karyotyping, which allowed the microscopic visualization of large-scale chromosomal anomalies—has now expanded into a multidimensional and high-resolution genomic landscape, powered by advanced molecular and imaging tools. Modern cytogenomic diagnostics integrate structural, functional, and spatial data, providing not only a linear sequence-based view of the genome, but also insights into its three-dimensional (3D) nuclear organization, epigenetic regulation, and dynamic chromatin behavior [83,84,85]. This chapter provides an overview of the main diagnostic technologies, highlighting their features, potential, and limitations in the modern clinical context.

### 5.1. Classical Cytogenetics: Principles, Applications, and Limitations

Classical cytogenetics form the historical foundation of chromosomal diagnosis, based on the microscopic observation of chromosomes during the metaphase using staining and banding techniques (G-, R-, C-banding) [86,87]. These methods allow the mapping of chromosome positions and the identification of gross structural rearrangements such as translocations, deletions, duplications, and inversions, with a resolution of several megabases.

A particularly relevant aspect is the link between chromosomal bands and functional genomic properties (Table 2), such as gene density and replication timing: gene-rich bands tend to replicate early in the S phase, while gene-poor bands replicate later, with significant diagnostic and biological implications [63,64]. This correlation between visible chromosomal structure and functional activity represents an initial bridge between cytogenetics and functional genomics.

This integrative perspective has led to the emergence of what we refer to as *functional cytogenomics*, a conceptual framework that extends classical cytogenetics by incorporating insights from gene expression, 3D genome architecture, and epigenetic features. The goal is to understand not only structural alterations, but also their downstream functional consequences. While similar concepts are discussed under the broader term chromosomics in some recent literature, we adopt functional cytogenomics here to highlight the diagnostic and mechanistic relevance of this integration in clinical settings.

However, the limited resolution (approximately 5–10 Mb) and dependence on sample quality and operator experience are significant limitations. These limitations have restricted the utility of classical cytogenetics in detecting complex or subtle chromosomal changes commonly seen in neurodevelopmental syndromes, recurrent miscarriage, and oncogenic transformation. For instance, balanced translocations or low-level mosaicisms may escape detection entirely. However, classical cytogenetics remain clinically indispensable, especially in settings such as prenatal diagnosis (e.g., trisomy 21, structural rearrangements), haematologic malignancies (e.g., chronic myeloid leukemia), and congenital anomaly syndromes, where large-scale chromosomal alterations are etiologically implicated. When integrated with molecular cytogenetics (e.g., FISH) or genomic platforms (e.g., array-CGH, WGS), classical cytogenetics contribute essential contextual and spatial information on genome architecture that complements high-resolution sequence data.

### 5.2. Fluorescence In Situ Hybridization: Increased Specificity and Sensitivity

The development of fluorescence in situ hybridization (FISH) marked a major milestone in cytogenetic diagnostics, significantly enhancing the ability to detect specific DNA sequences with higher resolution than classical banding techniques. Initially applied to metaphase chromosomes, FISH enabled the precise localization of DNA probes on intact chromosomal structures, overcoming the limitations of morphological interpretation and allowing the detection of submicroscopic rearrangements with a resolution around 100 kb [88,89].

The subsequent adaptations of the technique allowed hybridization on interphase nuclei, enabling the analysis of non-dividing cells and facilitating faster turnaround in clinical diagnostics. This adaptation proved particularly valuable in prenatal diagnostics, hematological malignancies, and solid tumors, where the rapid, cell-cycle-independent detection of chromosomal abnormalities is crucial [88,90,91]. A further innovation was the development of FISH on extended chromatin fibers (fiber-FISH), which dramatically increased the mapping resolution to the kilobase range and allowed the visualization of the fine-scale genomic architecture [92,93,94,95].

Multicolor FISH (mFISH) and spectral karyotyping (SKY), which use combinatorial labeling of multiple chromosome-specific probes, represented another breakthrough by enabling the simultaneous visualization of all chromosomes in distinct colors. These techniques significantly improved the detection of complex karyotypic abnormalities, such as cryptic translocations, insertions, or marker chromosomes, which are particularly relevant in cancer cytogenetics [92,96].

In oncology, FISH has become an essential diagnostic tool, not only for confirming structural rearrangements such as *BCR::ABL1*, *MYC* or *ETV6::RUNX1* fusions, but also for detecting gene amplifications (e.g., *HER2* in breast cancer) and chromosomal gains or losses that impact treatment decisions. Moreover, nuclear FISH provides insights into the spatial organization of chromosomal territories and its perturbation in malignant cells, linking genomic instability with nuclear architecture alterations [77].

Despite the emergence of high-throughput sequencing technologies, FISH remains a gold standard in targeted diagnostics due to its specificity, relatively low cost, and ability to detect known alterations directly in single cells. Its versatility, from metaphase spreads to interphase nuclei and fiber preparations, ensures its continued relevance in both research and clinical applications. Its continued utility spans both clinical applications and basic research, making FISH a powerful tool in modern cytogenomics.

### 5.3. High-Resolution Genomic Technologies: Array-CGH, SNP Arrays, and Optical Mapping

The advent of array-based technologies represented a further leap in resolution and genome-wide detection capacity following the development of FISH. Array-comparative genomic hybridization (array-CGH) and single nucleotide polymorphism (SNP) arrays allow for the high-throughput detection of submicroscopic copy number variations (CNVs), gains, losses, and loss of heterozygosity (LOH), with resolutions down to a few kilobases [97]. Unlike conventional cytogenetic approaches, these platforms do not require actively dividing cells, allowing their use on a broader range of biological samples and making them first-tier diagnostic tools in several clinical contexts.

Array-CGH employs the hybridization of fluorescently labeled genomic DNA onto oligonucleotide or BAC arrays covering the entire genome, while SNP arrays incorporate probes targeting polymorphic loci, enabling the detection of allelic imbalances, regions of homozygosity, and uniparental disomy (UPD) in addition to CNVs. Their application has led to the identification of pathogenic CNVs in patients with developmental disorders, neuropsychiatric conditions, and congenital malformations, often revealing alterations not detectable by conventional cytogenetics or FISH [98]. In oncology, arrays are extensively used for tumor genome profiling, identifying chromosomal imbalances associated with prognosis, therapeutic response, and risk stratification, such as *MYCN* amplification in neuroblastoma or 1p/19q co-deletion in gliomas, both of which carry diagnostic and therapeutic significance [99].

Moreover, array-CGH data have elucidated the impact of structural variants on topologically associating domain (TAD) boundaries, highlighting the functional consequences of CNVs on 3D genome organization and gene regulation [100]. This underscores a growing convergence between cytogenomics and functional epigenomics.

Optical mapping represents a newer frontier, allowing the direct analysis of ultra-long DNA molecules (>150 kb) through the incorporation of fluorescent tags at specific sequence motifs, followed by imaging. This technique provides highly accurate structural variant detection, even across repetitive or poorly mappable regions, making it a valuable complement to short-read sequencing and a promising tool in the diagnosis of complex rearrangements in both rare diseases and cancer [101]. Recent clinical applications have confirmed its diagnostic power, with optical mapping successfully identifying constitutional chromosomal aberrations in cases unresolved by conventional cytogenetics [102]. In this context, digital chromosomal imaging can be considered a form of ultra-high-resolution banding, in which each “band” corresponds to a defined nucleotide sequence, unlike classical banding methods, where the underlying sequences are more heterogeneous.

Despite their high resolution, array-based methods lack the ability to detect balanced rearrangements (such as inversions or translocations), due to the absence of copy number change. This limitation can be addressed by combining arrays with complementary techniques such as FISH or next-generation sequencing (NGS). In contrast, optical mapping enables the detection of both balanced and unbalanced events in a single assay, positioning it as a powerful addition to the cytogenomic diagnostic arsenal.

### 5.4. Genomic Sequencing: Short-Read and Long-Read

The transition from targeted genetic tests to genome-wide sequencing has marked a paradigm shift in clinical diagnostics. Next-generation sequencing (NGS) platforms have enabled the comprehensive analysis of the genome at base pair resolution, surpassing the limitations of hybridization-based approaches and offering the ability to detect small-scale sequence variants across the entire genome.

Short-read sequencing technologies, such as those developed by Illumina, generate large volumes of high-quality sequence data by reading DNA fragments of 100–300 base pairs. These platforms are highly effective for identifying point mutations, small insertions and deletions (indels), and micro-CNVs, and are now routinely widely used in clinical exome sequencing (CES) and whole-genome sequencing (WGS) workflows [103]. However, due to their limited read length, short-read technologies face challenges in accurately mapping repetitive or low-complexity regions, resolving complex structural variants (SVs), and identifying balanced rearrangements such as inversions and translocations.

To overcome these limitations, long-read sequencing technologies have emerged as a powerful complement to traditional NGS. Platforms—such as Pacific Biosciences (PacBio) Single Molecule Real-Time (SMRT) sequencing and Oxford Nanopore Technology (ONT)—offer a transformative advantage in resolving structural variation, spanning long genomic repeats, and accurately detecting balanced rearrangements such as inversions and translocations [104,105]. Reads exceeding tens of kilobases in length allow the direct phasing of haplotypes, improved genome assembly, and a more precise delineation of breakpoint junctions, which are particularly relevant in oncogenomics and rare disease diagnosis.

These platforms are also instrumental in elucidating the effects of genetic variation on higher-order chromatin architecture. By integrating sequencing data with cytogenetic maps and 3D genome models, it is now possible to assess not only the presence of a variant but also its potential disruption of regulatory landscapes, chromatin loops, and TAD boundaries [106]. This convergence of sequencing, cytogenomics, and nuclear organization is paving the way toward more comprehensive and functionally informed diagnostic interpretations.

Despite showing promise, long-read sequencing technologies are not without limitations related to sequencing error rates (especially for ONT), data storage, and cost efficiency. ONT, while capable of ultra-long reads, historically exhibits higher per-read error rates, although accuracy has improved significantly with recent chemistry and basecalling advances. Both the ONT and PacBio platforms entail substantial computational and data storage demands, and remain more cost intensive than short-read sequencing, although prices continue to decline [107]. To harness the complementary strengths of both platforms, hybrid strategies that combine short- and long-read data are increasingly used to leverage the strengths of both platforms, combining the high base accuracy of short reads with the structural resolution of long reads to optimize variant detection—particularly in cases involving complex rearrangements, repeat expansions, or mosaicism [107,108].

In conclusion, next-generation sequencing technologies, both short- and long-read, are integral to the modern cytogenomic diagnostic workflow. Their integration with classical cytogenetic methods and emerging tools like optical genome mapping supports a multimodal approach to genome analysis [108].

### 5.5. Current Limitations and Clinical Applicability of Cytogenomic Technologies

While cytogenomic technologies have greatly advanced the detection of structural variations, each method presents specific strengths and limitations that shape its clinical applicability. Karyotyping remains useful for detecting large chromosomal rearrangements, while FISH offers a targeted resolution. CMA provides genome-wide CNV detection but misses balanced events. Short-read sequencing offers broad coverage but struggles with complex SVs, whereas long-read sequencing improves structural resolution at a higher cost. Optical genome mapping excels at identifying large, complex rearrangements but lacks single-nucleotide resolution.

The main features, diagnostic applications, and limitations of the technologies discussed above are summarized in Table 3, highlighting the complementarity between classical cytogenetic and modern genomic approaches.

## 6. Cytogenomics in Cancer Diagnosis and Prognosis

### 6.1. Chromosomal Rearrangements in Cancer Diagnosis and Prognosis

Cytogenetics and cytogenomics have historically been pivotal in understanding cancer biology, offering key diagnostic and prognostic insights [85]. Chromosomal rearrangements, including deletions, duplications, translocations, and inversions, can result in the formation of chimeric genes or the deregulation of oncogenes and tumor suppressor genes. These alterations not only define specific tumor entities but also provide valuable information for risk stratification, treatment response, and disease monitoring [109].

In hematological malignancies, chromosomal rearrangements are critical diagnostic markers and are integral to the World Health Organization (WHO) classification. For example, the t(9;22)(q34;q11) results in the *BCR::ABL1* fusion gene. This fusion protein encodes a constitutively active tyrosine kinase, making it an ideal therapeutic target for tyrosine kinase inhibitors (TKIs) such as imatinib, which has dramatically improved survival rates [110]. Similarly, acute promyelocytic leukemia (APL) is defined by the t(15;17)(q24;q21) translocation generating the *PML::RARA* fusion gene. This fusion disrupts retinoic acid signaling and blocks myeloid differentiation. Importantly, it renders the disease highly responsive to differentiation therapy using all-trans retinoic acid (ATRA) in combination with arsenic trioxide, effectively transforming what was once a highly fatal leukemia into a largely curable disease [111]. Several other recurrent alterations, such as *ETV6::RUNX1*, *KMT2A* rearrangements, and high hyperdiploidy in childhood acute lymphoblastic leukemia, are associated with prognosis and treatment decisions. For instance, *ETV6::RUNX1* is associated with a favorable prognosis, whereas *KMT2A* fusions or hypodiploidy are typically linked to adverse outcomes [112].

Unbalanced SVs such as microdeletions and microduplications are also responsible for well-defined genomic syndromes. For instance, a 7q11.23 deletion results in Williams–Beuren syndrome, characterized by cardiovascular anomalies, intellectual disability, and distinctive facial features. Conversely, a 17p12 duplication encompassing the *PMP*22 gene causes Charcot–Marie–Tooth disease type 1A, the most common hereditary neuropathy [84]. These examples illustrate how SV-mediated gene dosage and regulatory disruption contribute to diverse clinical phenotypes.

### 6.2. Advances in Cytogenomic Technologies and Their Clinical Impact

Several of these structural anomalies have well-established roles in diagnosis, prognosis, and therapy across diverse cancer types. Table 4 summarizes key cytogenomic alterations, their molecular mechanisms, and clinical implications.

Solid tumors also display recurrent cytogenomic alterations, although their characterization is often more complex due to tumor heterogeneity and lower proliferation rates in vitro. Nevertheless, FISH and microarray-based technologies have contributed to the identification of clinically relevant markers, such as *ERBB2 (HER2)* amplification in breast cancer, which predicts the response to anti-HER2 therapies such as trastuzumab, or 1p/19q co-deletion in oligodendrogliomas, which inform both the prognosis and therapeutic response [122,123].

The advent of high-resolution and genome-wide techniques, including array comparative genomic hybridization (aCGH), single nucleotide polymorphism (SNP) arrays, and next-generation sequencing (NGS), has revolutionized cancer cytogenomics. These tools enable the detection of submicroscopic copy number variations, loss of heterozygosity, chromothripsis, and complex structural rearrangements that may escape conventional karyotyping [124,125]. Furthermore, the integration of cytogenomic data with transcriptomic, epigenomic, and proteomic datasets enables multi-dimensional tumor profiling, refining tumor classification systems and uncovering novel oncogenic drivers, tumor suppressor alterations, and regulatory disruptions. This approach, as exemplified by large-scale initiatives like the Pan-Cancer Analysis of Whole Genomes (PCAWG), has elucidated common and tissue-specific patterns of genomic instability, mutation timing, and structural variant architecture across diverse tumor types [126].

Emerging technologies such as single-cell genomics, spatial transcriptomics, and integrated multi-omics are poised to further revolutionize solid tumor diagnostics. These platforms allow the dissection of cellular subclones, the tracking of tumor evolution, and the exploration of cell–cell interactions within the tumor microenvironment—all of which are critical for understanding therapeutic resistance, immune evasion, and metastasis. Together, these technological advances underscore the pivotal role of cytogenomics in driving personalized cancer diagnostics and therapeutics.

## 7. Integrating 3D Genome and Epigenome Data in Clinical Contexts

The integration of 3D genome organization data with epigenomic information represents an emerging paradigm in clinical diagnostics, providing a functional understanding of genomic alterations and their pathological implications, with direct relevance to clinical decision making [127,128].

### 7.1. Emerging Techniques for 3D Genome and Epigenomic Profiling

Hi-C and its variants (e.g., Capture Hi-C, Micro-C) enable the mapping of chromatin interactions on a genome-wide or targeted scale and enable genome-wide or locus-specific mapping of chromatin interactions, revealing the hierarchical organization of DNA into topologically associating domains (TADs), chromatin loops, and nuclear compartments (A/B) [19,20,129]. These data clarify long-range regulatory interactions, especially enhancer–promoter contacts. Capture Hi-C, in particular, enables the targeted analysis of disease-relevant loci identified by GWAS or structural variant studies [129].

Concurrently, ChIP-seq (Chromatin Immunoprecipitation sequencing) provides a genome-wide map of DNA–protein interactions, particularly histone modifications (e.g., H3K27ac for active enhancers, H3K27me3 for repressed regions) or the binding of key transcription factors, outlining the epigenetic landscape regulating gene expression. ChIP-seq is essential for identifying the regulatory elements that orchestrate cell type-specific gene expression programs [130,131].

ATAC-seq (Assay for Transposase-Accessible Chromatin using sequencing) complements ChIP-seq by identifying open chromatin regions across the genome. These regions are typically nucleosome-depleted and accessible to transcription factors, serving as potential regulatory elements such as enhancers, promoters, and insulators [132]. ATAC-seq requires minimal input material and provides rapid profiling of chromatin accessibility, making it well suited for both basic and clinical research.

When integrated, these multi-omic approaches allow the reconstruction of 3D regulatory networks, which is essential for understanding how structural variants or epigenetic alterations disrupt genome function. Such disruptions can lead to enhancer hijacking, TAD boundary disruption, and gene misexpression, particularly in cancer and developmental disorders [6,133]. These technologies are increasingly incorporated into clinical genomics and disease modeling pipelines, helping to reveal mechanistic insights into how genetic and epigenetic changes influence phenotypic outcomes. A comparison of the available technologies is detailed in Table 5.

### 7.2. Examples of Clinical Applications

A paradigmatic example is enhancer hijacking, where chromosomal rearrangements relocate enhancers near oncogenes, such as *MYC* or *TERT*, leading to their ectopic activation without gene mutation [27,118]. Similar principles apply to congenital disorders like craniofacial and cardiac malformations, where TAD disruption displaces regulatory elements, altering developmental gene expression despite unmutated coding sequences [134,135].

3D genome mapping has uncovered the molecular basis of such conditions, offering new diagnostic and therapeutic opportunities. This shift highlights how pathogenicity can arise from structural alterations in non-coding regions that rewire regulatory interactions. As a result, chromatin topology analysis is becoming integral to clinical genomics, revealing hidden disease mechanisms and guiding targeted interventions based on spatial genome organization.

### 7.3. Towards Integrated Functional Cytogenomics

To translate these complex datasets into actionable insights, robust bioinformatic pipelines are essential. The integration of structural variation, epigenomic, and transcriptomic data requires sophisticated computational tools capable of managing and interpreting multi-layered information. Public databases such as ENCODE, 4D Nucleome, and Roadmap Epigenomics provide essential resources for contextualizing clinical data and comparing patient profiles to control populations [136,137].

The growing application of artificial intelligence and machine learning algorithms has improved the classification of complex structural variants and the prediction of the functional impact of 3D alterations, facilitating clinical interpretation [138,139,140]. These tools are currently under development to become integral components of future diagnostic and prognostic frameworks.

### 7.4. Future Perspectives

Looking ahead, integrated “functional cytogenomics” with multi-omics data will be a powerful tool for precision medicine, capable of enabling the definition of patient-specific molecular signatures and guiding targeted therapies based not only on genetic mutations but also on alterations in the nuclear architecture and epigenetic landscape [7,128]. The continued advancement of single-cell sequencing technologies, in conjunction with three-dimensional chromatin conformation analyses, will open new frontiers in our understanding of cellular heterogeneity. These developments will be particularly valuable in dissecting the cellular complexity of multifactorial diseases and identifying novel biomarkers for disease subtypes and therapeutic response [141,142].

## 8. Challenges and Future Perspectives

The integrated analysis of 3D genome structure and its epigenetic modifications is rapidly emerging as a cornerstone in the clinical diagnostics of genetic and neoplastic diseases. The ability to map chromatin interactions, topological domains, and epigenetic states provides a functional perspective that transcends linear DNA sequence information [6,143]. It allows for the identification of regulatory mechanisms underlying gene expression control, chromatin compartmentalization, and genome stability.

Recent technological advances—such as genome-wide and single-cell sequencing combined with high-resolution cytogenetic methods—are enabling the multidimensional integration of genomic, epigenomic, and transcriptomic data [144,145]. This holistic view facilitates not only the identification of point mutations or structural rearrangements but also the detection of disruptions in the spatial and temporal regulation of gene expression, which can underlie complex pathological phenotypes [21,127].

In clinical practice, the incorporation of nuclear organization studies into diagnostic workflows—alongside traditional genetic testing—holds the potential to refine tumor classification, enable the early detection of functional disruptions, and support therapy stratification. It may improve tumor subclassification, enable the earlier detection of regulatory disruptions, and facilitate the stratification of patients for targeted therapeutic interventions. Importantly, these spatial genomic features can serve as biomarkers for disease progression and therapeutic response, especially in cancers and neurodevelopmental disorders.

However, several challenges remain. These include the standardization of protocols, the reduction in implementation costs, and the development of scalable bioinformatic tools capable of processing and interpreting complex, multidimensional datasets. Equally important is the multidisciplinary training of healthcare and laboratory professionals, which will be essential to ensure the effective adoption of these innovations in routine diagnostics [146,147,148].

Future directions are promising: the convergence of multi-omics technologies and artificial intelligence is expected to yield highly personalized predictive models that integrate 3D genome architecture with environmental and clinical data [149,150]. These developments will lay the groundwork for more refined and timely interventions in genetic, oncological, and neurodegenerative diseases.

## 9. Conclusions

The integration of 3D genome architecture and epigenetic modifications into clinical diagnostics represents a paradigm shift in our understanding and management of genetic and neoplastic disorders. This multidimensional approach goes beyond linear DNA sequence analysis, revealing higher-order mechanisms that regulate gene expression and genome stability.

Advances in genome-wide and single-cell technologies, combined with advanced cytogenetic approaches, have already begun to impact clinical practice—particularly in oncology, where chromatin topology contributes to disease stratification and therapeutic decision making. Notably, cytogenetic approaches often retain greater predictive value than gene mutation profiling alone, particularly when structural context is critical.

Nevertheless, challenges remain. These include the standardization of experimental and analytical protocols, the need for cost-effective implementation, and the development of robust bioinformatic platforms tailored to clinical use. Interdisciplinary training will play a pivotal role in bridging the gap between innovation and clinical applicability.

Practical implementation requires context-driven decisions: optical genome mapping (OGM) is optimal for unresolved structural variants in haematologic malignancies or complex constitutional rearrangements; whole-genome sequencing (WGS) suits undiagnosed genetic disorders; chromosomal microarrays (CMA) remain first-line for neurodevelopmental phenotypes; and long-read sequencing offers resolution for repeat expansions or complex SVs.

To guide clinical application, we propose a tiered approach: CMA for the initial screening of developmental anomalies; WGS for comprehensive variant detection in undiagnosed cases; OGM for complex structural variants, particularly in oncology; and long-read sequencing for resolving challenging structural variants and repeat expansions. This strategy balances resolution, cost, and turnaround time to optimize diagnostic efficiency.

These decisions should be guided by clinical presentation, resolution needs, and resource availability, supporting a more personalized and efficient diagnostic workflow.

Finally, understanding genome architecture also benefits from evolutionary frameworks such as the Genome Architecture Theory, which emphasizes the regulatory role of karyotype coding in maintaining genome stability and cell identity. This perspective is particularly relevant in studying macroevolution, where chromosomal alterations, rather than single gene mutations, are recognized as key drivers of genomic innovation and adaptation.

Looking ahead, the integration of multi-omics approaches with artificial intelligence holds great promise for revolutionizing precision medicine. These integrative frameworks will enable the construction of highly individualized predictive models that incorporate not only genomic and epigenomic features, but also environmental exposures, clinical phenotypes, and temporal dynamics. Such models have the potential to drastically improve diagnostic accuracy, enable the early detection of disease states, guide personalized therapeutic regimens, and ultimately enhance clinical outcomes across a broad spectrum of genetic, oncologic, and neurodegenerative disorders.

In summary, embracing the complexity of genome architecture and integrating cytogenomic insights into routine diagnostics is no longer optional but imperative for the next leap in personalized medicine. Concerted efforts among researchers, clinicians, and bioinformaticians to standardize methodologies, expand interdisciplinary training, and foster collaborative innovation will accelerate the translation of these advances into tangible patient benefits. By doing so, we pave the way for a future where diagnosis and treatment are precisely tailored not only to the genetic code but also to the dynamic spatial and regulatory genome landscape, fundamentally transforming healthcare outcomes.

## Figures and Tables

**Table 1:** Major mechanisms of chromosomal rearrangement and their clinical implications.

Mechanism	Description	Types of Rearrangements	Associated Disorders
Non-Homologous End Joining (NHEJ)	Error-prone repair of DNA double-strand breaks without homology	Translocations, deletions, insertions	Leukaemias (e.g., *BCR::ABL1*), lymphomas
Microhomology-Mediated End Joining (MMEJ)	Alternative end joining using short homologous sequences (microhomologies)	Small deletions and complex rearrangements	Genetic syndromes with structural variation
Fork Stalling and Template Switching (FoSTeS)	Template switching during replication fork stalling	Complex duplications and deletions	Genomic disorders (e.g., *MECP2* duplication syndrome)
Non-Allelic Homologous Recombination (NAHR)	Recombination between low copy repeats leading to misalignment	Recurrent deletions, duplications, inversions	DiGeorge syndrome (22q11.2), Williams syndrome
Chromothripsis	Massive chromosome fragmentation and disordered reassembly	Complex rearrangements	Aggressive solid tumors, neuroblastoma
Chromoanasynthesis	Replication-based mechanism involving serial template switching and fork collapse	Complex duplications, deletions, triplications	Neurodevelopmental disorders, congenital anomalies
Chromoplexy	Interdependent chromosomal translocations and deletions, often in a single event	Balanced and unbalanced rearrangements	Prostate cancer, other solid tumors

**Table 2:** Distinctive genomic features related to chromosomal bands and replication timing.

	Replication Timing ^(a)^ and Band Staining Properties ^(b)^				
Features	Very Early ^(a)^ R-Positive ^(b)^	Early ^(a)^ R-Positive ^(b)^	Late ^(a)^ R-Negative ^(b)^	Very Late ^(a)^ R-Negative ^(b)^	
Replication category ^(c)^	1–3	4–9	10–14	15–19	[64,72]
GC content	>52%	44–52%	37–44%	<37%	[74]
Gene density	>15 genes/Mb	5–15 genes/Mb	5–15 genes/Mb	<5 genes/Mb	[75,76]
General chromatin state	Highly decondensed	Variable ^(d)^	Variable ^(d)^	Highly condensed	[10,66]
General transcriptional activity	High	Variable ^(d)^	Variable ^(d)^	Low	[63]
Intranuclear localization	Internal	Intermediate	Intermediate	Peripheral	[14,77]
Sensitivity to replication stress	Low	Intermediate	Intermediate	High	[78]

^(a)^ DNA replicated before (early) or after (late) the BrdU-induced replication block. ^(b)^ Reverse banding patterns, based on heat treatment of chromosomes. ^(c)^ Band groups as defined by Dutrillaux et al. [72]. ^(d)^ Variability depending on the adjacent band type (very GC-rich or very-GC-poor).

**Table 3:** Diagnostic technologies in cytogenetics and cytogenomics: main characteristics and clinical applications.

Technique	Resolution	Type of Variant Detected	Main Applications	Main Limitations	Clinical Use
Classical Cytogenetics (Banding)	~5–10 Mb	Numerical abnormalities, large unbalanced or balanced rearrangements	Chromosomal syndromes; constitutional and acquired translocations	Low resolution; subjective interpretation; limited to dividing cells	Prenatal, hematology, infertility
FISH	~100 kb	Targeted deletions, duplications, translocations	Rapid targeted diagnosis; monitoring known aberrations	Not genome-wide; probe design required	Oncology, known microdeletions
Array-CGH	~10–50 kb	Submicroscopic copy number variations (CNVs)	Diagnosis of genomic syndromes; oncogenomic profiling	Cannot detect balanced rearrangements	Syndromic anomalies, prenatal testing
SNP Arrays	~10–50 kb	CNVs, copy-neutral LOH, uniparental disomy	Diagnosis of genetic diseases; tumor susceptibility screening	Limited for structural complexity; no balanced rearrangements	Oncology
Optical Mapping	kb–Mb	Complex rearrangements, large insertions, repeats	Cancer diagnostics; structural variant resolution in genetic disorders	Still emerging; requires high molecular weight DNA	Cancer, constitutional SV screening
Short-Read Sequencing	bp–kb	SNVs, indels, micro-CNVs	Exome/genome sequencing; detailed molecular diagnostics	Poor detection of structural variants in repetitive regions	Rare diseases, cancer genomics
Long-Read Sequencing	kb–Mb	Complex structural variants, repeat expansions, inversions	Rare disease diagnosis; structural variant characterization; research	High cost; error rates (platform-dependent); large data volume	Complex cases, unresolved diagnostics

**Table 4:** Representative cytogenomic alterations in cancer: underlying mechanisms and diagnostic/prognostic relevance.

Cytogenomic Alteration	Tumor Type	Mechanism	Clinical Implications	References
t(9;22)(q34;q11)—*BCR::ABL1*	Chronic myeloid leukemia	Balanced translocation and fusion gene	Diagnostic marker; targeted therapy with tyrosine kinase inhibitors	[113,114]
t(14;18)(q32;q21)—*BCL2*	Follicular lymphoma	Balanced translocation	Diagnostic and prognostic marker	[115,116]
Amplifications and rearrangements of *MYC*	Aggressive lymphomas, solid tumors	Amplifications and translocations	Poor prognosis; oncogenic activation	[117]
Chromothripsis	Solid and hematological malignancies	Complex genome rearrangement	Genomic instability; unfavorable prognosis	[41]
Enhancer hijacking	Leukaemias, solid tumors	Oncogene activation via aberrant enhancer contacts	Novel diagnostic and therapeutic targets	[7,118]
5q deletions	Myelodysplastic syndrome, acute leukemia	Genomic loss	Loss of tumor suppressor genes; prognostic significance	[119]
inv(16)(p13q22)	Acute myeloid leukemia	Chromosomal inversion and fusion gene	Favorable prognosis; diagnostic marker	[120]
del(17p)—*TP53*	Various tumors and leukaemias	Tumor suppressor gene loss	Poor prognosis; therapy resistance	[121]

**Table 5:** Technologies for 3D genome and epigenomic profiling.

Technique	Principle	Clinical Applications	Strengths	Limitations
Hi-C	Genome-wide sequencing of chromatin interactions	Detection of altered TADs, chromatin loops, and rearrangements in cancer and genetic diseases	Genome-wide coverage, 3D spatial information	Limited resolution without enrichment
Capture Hi-C	Targeted enrichment of Hi-C libraries	High-resolution analysis of disease-associated loci	Target specificity, high resolution	Dependent on prior selection of target regions
ChIP-seq	Sequencing of DNA bound to histone marks or TFs via immunoprecipitation	Epigenetic profiling in cancers and developmental disorders	Functional annotation of regulatory states	Requires specific antibodies and good-quality samples
ATAC-seq	Tagging of open chromatin with Tn5 transposase	Active regulatory region identification, tumor heterogeneity	Low input, high resolution	No protein-specific information
HiChIP/PLAC-seq	Combination of Hi-C and ChIP-seq to map protein-mediated chromatin contacts	Enhancer–promoter mapping in rare diseases and cancer	Protein-centric 3D contact profiling	Technically complex and expensive
Single-cell Hi-C/ATAC-seq	Cell-level profiling of 3D structure or chromatin accessibility	Heterogeneity analysis in cancer and rare disease	Single-cell resolution, fine population analysis	High cost, complex data analysis
DNA FISH	Fluorescent hybridization of DNA probes on cells or nuclei	Rearrangement detection, nuclear positioning analysis in cancers	Visual validation, widely used in diagnostics	Limited resolution, not genome wide
CUT&Tag/CUT&RUN	Epigenetic profiling with minimal material input	Rapid profiling in clinical specimens	Low input requirements, high sensitivity	Protocol standardization still required for clinical use
Optical Mapping	High-resolution physical mapping of long DNA molecules	Detection of complex rearrangements, integration with 3D genome data	Complementary to sequencing, provides structural insight	Lacks direct 3D spatial data

## Abbreviations

The following abbreviations are used in this manuscript:

3C	Chromosome Conformation Capture
3D	Three-dimensional
4C	Circular Chromosome Conformation Capture
5C	Carbon Copy Chromosome Conformation Capture
AI	Artificial Intelligence
ALL	Acute Lymphoblastic Leukemia
AML	Acute Myeloid Leukemia
APL	Acute Promyelocytic Leukemia
ATAC-seq	Assay for Transposase-Accessible Chromatin using sequencing
ATRA	All-Trans Retinoic Acid
B-ALL	B-cell Acute Lymphoblastic Leukemia
CGH	Comparative Genomic Hybridization
ChIP-seq	Chromatin Immunoprecipitation sequencing
CLL	Chronic Lymphocytic Leukemia
CMA	Chromosomal Microarrays
CML	Chronic Myeloid Leukemia
CNS	Central Nervous System
CNV	Copy Number Variant
CRISPR	Clustered Regularly Interspaced Short Palindromic Repeats
DAPI	4′,6-diamidino-2-phenylindole
DMR	Differentially Methylated Region
ESC	Embryonic Stem Cell
FISH	Fluorescence In Situ Hybridization
FoSTeS	Fork Stalling and Template Switching
FRET	Förster Resonance Energy Transfer
G-banding	Giemsa banding
GWAS	Genome-Wide Association Study
H&E	Hematoxylin and Eosin
H3K27me3	Histone 3 Lysine 27 trimethylation
HDAC	Histone Deacetylase
Hi-C	High-throughput Chromosome Conformation Capture
ICF syndrome	Immunodeficiency, Centromeric instability, and Facial anomalies syndrome
iPSC	Induced Pluripotent Stem Cell
LOH	Loss of Heterozygosity
MDS	Myelodysplastic Syndromes
MMEJ	Microhomology-Mediated End Joining
NAHR	Non-Allelic Homologous Recombination
NHEJ	Non-Homologous End Joining
OGM	Optical Genome Mapping
TAD	Topologically Associating Domain
TERT	Telomerase Reverse Transcriptase
TF	Transcription Factor
WES	Whole-Exome Sequencing
WGS	Whole-Genome Sequencing

## References

[1] Balciuniene J., Ning Y., Lazarus H.M., Aikawa V., Sherpa S., Zhang Y., Morrissette J.J.D. (2024). Cancer Cytogenetics in a Genomics World: Wedding the Old with the New. Blood Rev..

[2] Liehr T. (2010). Cytogenetic Contribution to Uniparental Disomy (UPD). Mol. Cytogenet..

[3] Alkan C., Coe B.P., Eichler E.E. (2011). Genome Structural Variation Discovery and Genotyping. Nat. Rev. Genet..

[4] Collins R.L., Brand H., Redin C.E., Hanscom C., Antolik C., Stone M.R., Glessner J.T., Mason T., Pregno G., Dorrani N. (2017). Defining the Diverse Spectrum of Inversions, Complex Structural Variation, and Chromothripsis in the Morbid Human Genome. Genome Biol..

[5] Federico C., Bruno F., Ragusa D., Clements C.S., Brancato D., Henry M.P., Bridger J.M., Tosi S., Saccone S. (2021). Chromosomal Rearrangements and Altered Nuclear Organization: Recent Mechanistic Models in Cancer. Cancers.

[6] Bonev B., Cavalli G. (2016). Organization and Function of the 3D Genome. Nat. Rev. Genet..

[7] Spielmann M., Lupiáñez D.G., Mundlos S. (2018). Structural Variation in the 3D Genome. Nat. Rev. Genet..

[8] Van Steensel B., Furlong E.E.M. (2019). The Role of Transcription in Shaping the Spatial Organization of the Genome. Nat. Rev. Mol. Cell Biol..

[9] Cremer T., Cremer M., Hübner B., Strickfaden H., Smeets D., Popken J., Sterr M., Markaki Y., Rippe K., Cremer C. (2015). The 4D Nucleome: Evidence for a Dynamic Nuclear Landscape Based on Co-aligned Active and Inactive Nuclear Compartments. FEBS Lett..

[10] Saccone S., Federico C., Bernardi G. (2002). Localization of the Gene-Richest and the Gene-Poorest Isochores in the Interphase Nuclei of Mammals and Birds. Gene.

[11] Lanctôt C., Cheutin T., Cremer M., Cavalli G., Cremer T. (2007). Dynamic Genome Architecture in the Nuclear Space: Regulation of Gene Expression in Three Dimensions. Nat. Rev. Genet..

[12] Bickmore W.A. (2013). The Spatial Organization of the Human Genome. Annu. Rev. Genom. Hum. Genet..

[13] Maass P.G., Barutcu A.R., Rinn J.L. (2019). Interchromosomal Interactions: A Genomic Love Story of Kissing Chromosomes. J. Cell Biol..

[14] Federico C., Cantarella C.D., Di Mare P., Tosi S., Saccone S. (2008). The Radial Arrangement of the Human Chromosome 7 in the Lymphocyte Cell Nucleus Is Associated with Chromosomal Band Gene Density. Chromosoma.

[15] Daban J. (2024). Rethinking Models of DNA Organization in Micrometer-Sized Chromosomes from the Perspective of the Nanoproperties of Chromatin Favoring a Multilayer Structure. Small Struct..

[16] Ye C.J., Stilgenbauer L., Moy A., Liu G., Heng H.H. (2019). What Is Karyotype Coding and Why Is Genomic Topology Important for Cancer and Evolution?. Front. Genet..

[17] Heng J., Heng H.H. (2021). Karyotype Coding: The Creation and Maintenance of System Information for Complexity and Biodiversity. Biosystems.

[18] Furst R. (2021). The Importance of Henry H. Heng’s Genome Architecture Theory. Prog. Biophys. Mol. Biol..

[19] Dixon J.R., Selvaraj S., Yue F., Kim A., Li Y., Shen Y., Hu M., Liu J.S., Ren B. (2012). Topological Domains in Mammalian Genomes Identified by Analysis of Chromatin Interactions. Nature.

[20] Lieberman-Aiden E., Van Berkum N.L., Williams L., Imakaev M., Ragoczy T., Telling A., Amit I., Lajoie B.R., Sabo P.J., Dorschner M.O. (2009). Comprehensive Mapping of Long-Range Interactions Reveals Folding Principles of the Human Genome. Science.

[21] Rao S.S.P., Huntley M.H., Durand N.C., Stamenova E.K., Bochkov I.D., Robinson J.T., Sanborn A.L., Machol I., Omer A.D., Lander E.S. (2014). A 3D Map of the Human Genome at Kilobase Resolution Reveals Principles of Chromatin Looping. Cell.

[22] Naumova N., Imakaev M., Fudenberg G., Zhan Y., Lajoie B.R., Mirny L.A., Dekker J. (2013). Organization of the Mitotic Chromosome. Science.

[23] Cremer T., Cremer M., Hübner B., Silahtaroglu A., Hendzel M., Lanctôt C., Strickfaden H., Cremer C. (2020). The Interchromatin Compartment Participates in the Structural and Functional Organization of the Cell Nucleus. BioEssays.

[24] Ballabio E., Cantarella C.D., Federico C., Di Mare P., Hall G., Harbott J., Hughes J., Saccone S., Tosi S. (2009). Ectopic Expression of the *HLXB9* Gene Is Associated with an Altered Nuclear Position in t(7;12) Leukaemias. Leukemia.

[25] Federico C., Pappalardo A.M., Ferrito V., Tosi S., Saccone S. (2017). Genomic Properties of Chromosomal Bands Are Linked to Evolutionary Rearrangements and New Centromere Formation in Primates. Chromosome Res..

[26] Federico C., Brancato D., Bruno F., Galvano D., Caruso M., Saccone S. (2024). Robertsonian Translocation between Human Chromosomes 21 and 22, Inherited across Three Generations, without Any Phenotypic Effect. Genes.

[27] Flavahan W.A., Drier Y., Liau B.B., Gillespie S.M., Venteicher A.S., Stemmer-Rachamimov A.O., Suvà M.L., Bernstein B.E. (2016). Insulator Dysfunction and Oncogene Activation in *IDH* Mutant Gliomas. Nature.

[28] Tillotson R., Bird A. (2020). The Molecular Basis of MeCP2 Function in the Brain. J. Mol. Biol..

[29] Lammerding J., Schulze P.C., Takahashi T., Kozlov S., Sullivan T., Kamm R.D., Stewart C.L., Lee R.T. (2004). Lamin A/C Deficiency Causes Defective Nuclear Mechanics and Mechanotransduction. J. Clin. Investig..

[30] Bridger J.M., Foeger N., Kill I.R., Herrmann H. (2007). The Nuclear Lamina: Both a Structural Framework and a Platform for Genome Organization. FEBS J..

[31] Shimi T., Butin-Israeli V., Adam S.A., Hamanaka R.B., Goldman A.E., Lucas C.A., Shumaker D.K., Kosak S.T., Chandel N.S., Goldman R.D. (2011). The Role of Nuclear Lamin B1 in Cell Proliferation and Senescence. Genes. Dev..

[32] Camps J., Erdos M.R., Ried T. (2015). The Role of Lamin B1 for the Maintenance of Nuclear Structure and Function. Nucleus.

[33] Lyst M.J., Bird A. (2015). Rett Syndrome: A Complex Disorder with Simple Roots. Nat. Rev. Genet..

[34] Brancato D., Bruno F., Coniglio E., Sturiale V., Saccone S., Federico C. (2024). The Chromatin Organization Close to SNP Rs12913832, Involved in Eye Color Variation, Is Evolutionary Conserved in Vertebrates. Int. J. Mol. Sci..

[35] Visser M., Kayser M., Grosveld F., Palstra R. (2014). Genetic Variation in Regulatory DNA Elements: The Case of *OCA 2* Transcriptional Regulation. Pigment. Cell Melanoma Res..

[36] Misteli T. (2020). The Self-Organizing Genome: Principles of Genome Architecture and Function. Cell.

[37] Deng S., Feng Y., Pauklin S. (2022). 3D Chromatin Architecture and Transcription Regulation in Cancer. J. Hematol. Oncol..

[38] Feuk L., Carson A.R., Scherer S.W. (2006). Structural Variation in the Human Genome. Nat. Rev. Genet..

[39] Lieber M.R. (2008). The Mechanism of Human Nonhomologous DNA End Joining. J. Biol. Chem..

[40] Zhang F., Gu W., Hurles M.E., Lupski J.R. (2009). Copy Number Variation in Human Health, Disease, and Evolution. Annu. Rev. Genom. Hum. Genet..

[41] Stephens P.J., Greenman C.D., Fu B., Yang F., Bignell G.R., Mudie L.J., Pleasance E.D., Lau K.W., Beare D., Stebbings L.A. (2011). Massive Genomic Rearrangement Acquired in a Single Catastrophic Event during Cancer Development. Cell.

[42] Plaisancié J., Kleinfinger P., Cances C., Bazin A., Julia S., Trost D., Lohmann L., Vigouroux A. (2014). Constitutional Chromoanasynthesis: Description of a Rare Chromosomal Event in a Patient. Eur. J. Med. Genet..

[43] Pellestor F., Ganne B., Gaillard J.B., Gatinois V. (2025). Chromoplexy: A Pathway to Genomic Complexity and Cancer Development. Int. J. Mol. Sci..

[44] Sakofsky C.J., Malkova A. (2017). Break Induced Replication in Eukaryotes: Mechanisms, Functions, and Consequences. Crit. Rev. Biochem. Mol. Biol..

[45] Stankiewicz P., Lupski J.R. (2010). Structural Variation in the Human Genome and Its Role in Disease. Annu. Rev. Med..

[46] Hancks D.C., Kazazian H.H. (2016). Roles for Retrotransposon Insertions in Human Disease. Mob. DNA.

[47] Weischenfeldt J., Symmons O., Spitz F., Korbel J.O. (2013). Phenotypic Impact of Genomic Structural Variation: Insights from and for Human Disease. Nat. Rev. Genet..

[48] Botten G., Zhang Y., Dudnyk K., Kim Y.J., Liu X., Sanders J.T., Imanci A., Droin N.M., Cao H., Kaphle P. (2023). Structural Variation Cooperates with Permissive Chromatin to Control Enhancer Hijacking-Mediated Oncogenic Transcription. Blood.

[49] Lupiáñez D.G., Spielmann M., Mundlos S. (2016). Breaking TADs: How Alterations of Chromatin Domains Result in Disease. Trends Genet..

[50] Hehlmann R., Hochhaus A., Baccarani M. (2007). Chronic Myeloid Leukaemia. Lancet.

[51] Tallman M.S., Gilliland D.G., Rowe J.M. (2005). Drug Therapy for Acute Myeloid Leukemia. Blood.

[52] McDonald-McGinn D.M., Sullivan K.E., Marino B., Philip N., Swillen A., Vorstman J.A.S., Zackai E.H., Emanuel B.S., Vermeesch J.R., Morrow B.E. (2015). 22q11.2 Deletion Syndrome. Nat. Rev. Dis. Primers.

[53] Döhner H., Estey E., Grimwade D., Amadori S., Appelbaum F.R., Büchner T., Dombret H., Ebert B.L., Fenaux P., Larson R.A. (2017). Diagnosis and Management of AML in Adults: 2017 ELN Recommendations from an International Expert Panel. Blood.

[54] Cortés-Ciriano I., Lee J.J.-K., Xi R., Jain D., Jung Y.L., Yang L., Gordenin D., Klimczak L.J., Zhang C.-Z., Pellman D.S. (2020). Comprehensive Analysis of Chromothripsis in 2,658 Human Cancers Using Whole-Genome Sequencing. Nat. Genet..

[55] Molenaar J.J., Koster J., Zwijnenburg D.A., Van Sluis P., Valentijn L.J., Van Der Ploeg I., Hamdi M., Van Nes J., Westerman B.A., Van Arkel J. (2012). Sequencing of Neuroblastoma Identifies Chromothripsis and Defects in Neuritogenesis Genes. Nature.

[56] Brand H., Pillalamarri V., Collins R.L., Eggert S., O’Dushlaine C., Braaten E.B., Stone M.R., Chambert K., Doty N.D., Hanscom C. (2014). Cryptic and Complex Chromosomal Aberrations in Early-Onset Neuropsychiatric Disorders. Am. J. Hum. Genet..

[57] Miller D.T., Shen Y., Weiss L.A., Korn J., Anselm I., Bridgemohan C., Cox G.F., Dickinson H., Gentile J., Harris D.J. (2009). Microdeletion/Duplication at 15q13.2q13.3 among Individuals with Features of Autism and Other Neuropsychiatric Disorders. J. Med. Genet..

[58] Sedlazeck F.J., Rescheneder P., Smolka M., Fang H., Nattestad M., Von Haeseler A., Schatz M.C. (2018). Accurate Detection of Complex Structural Variations Using Single-Molecule Sequencing. Nat. Methods.

[59] Sima J., Gilbert D.M. (2014). Complex Correlations: Replication Timing and Mutational Landscapes during Cancer and Genome Evolution. Curr. Opin. Genet. Dev..

[60] Rivera-Mulia J.C., Gilbert D.M. (2016). Replication Timing and Transcriptional Control: Beyond Cause and Effect—Part III. Curr. Opin. Cell Biol..

[61] Marchal C., Sima J., Gilbert D.M. (2019). Control of DNA Replication Timing in the 3D Genome. Nat. Rev. Mol. Cell Biol..

[62] Du Z., Zheng H., Huang B., Ma R., Wu J., Zhang X., He J., Xiang Y., Wang Q., Li Y. (2017). Allelic Reprogramming of 3D Chromatin Architecture during Early Mammalian Development. Nature.

[63] Pope B.D., Ryba T., Dileep V., Yue F., Wu W., Denas O., Vera D.L., Wang Y., Hansen R.S., Canfield T.K. (2014). Topologically Associating Domains Are Stable Units of Replication-Timing Regulation. Nature.

[64] Federico C., Saccone S., Bernardi G. (1998). The Gene-Richest Bands of Human Chromosomes Replicate at the Onset of the S-Phase. Cytogenet. Genome Res..

[65] Ryba T., Hiratani I., Lu J., Itoh M., Kulik M., Zhang J., Schulz T.C., Robins A.J., Dalton S., Gilbert D.M. (2010). Evolutionarily Conserved Replication Timing Profiles Predict Long-Range Chromatin Interactions and Distinguish Closely Related Cell Types. Genome Res..

[66] Van Steensel B., Belmont A.S. (2017). Lamina-Associated Domains: Links with Chromosome Architecture, Heterochromatin, and Gene Repression. Cell.

[67] Mattarocci S., Shyian M., Lemmens L., Damay P., Altintas D.M., Shi T., Bartholomew C.R., Thomä N.H., Hardy C.F.J., Shore D. (2014). Rif1 Controls DNA Replication Timing in Yeast through the PP1 Phosphatase Glc7. Cell Rep..

[68] Foti R., Gnan S., Cornacchia D., Dileep V., Bulut-Karslioglu A., Diehl S., Buness A., Klein F.A., Huber W., Johnstone E. (2016). Nuclear Architecture Organized by Rif1 Underpins the Replication-Timing Program. Mol. Cell.

[69] Hiratani I., Ryba T., Itoh M., Yokochi T., Schwaiger M., Chang C.-W., Lyou Y., Townes T.M., Schübeler D., Gilbert D.M. (2008). Global Reorganization of Replication Domains During Embryonic Stem Cell Differentiation. PLoS Biol..

[70] Meuleman W., Peric-Hupkes D., Kind J., Beaudry J.-B., Pagie L., Kellis M., Reinders M., Wessels L., Van Steensel B. (2013). Constitutive Nuclear Lamina–Genome Interactions Are Highly Conserved and Associated with A/T-Rich Sequence. Genome Res..

[71] Koren A., Polak P., Nemesh J., Michaelson J.J., Sebat J., Sunyaev S.R., McCarroll S.A. (2012). Differential Relationship of DNA Replication Timing to Different Forms of Human Mutation and Variation. Am. J. Hum. Genet..

[72] Dutrillaux B., Couturier J., Richer C.-L., Viegas-Péquignot E. (1976). Sequence of DNA Replication in 277 R- and Q-Bands of Human Chromosomes Using a BrdU Treatment. Chromosoma.

[73] Costantini M., Bernardi G. (2008). Replication Timing, Chromosomal Bands, and Isochores. Proc. Natl. Acad. Sci. USA.

[74] Bernardi G. (2005). Structural and Evolutionary Genomics: Natural Selection in Genome Evolution.

[75] Saccone S., Pavliček A., Federico C., Pačes J., Bernardi G. (2001). Genes, Isochores and Bands in Human Chromosomes 21 and 22. Chromosome Res..

[76] Lander E.S., Linton L.M., Birren B., Nusbaum C., Zody M.C., Baldwin J., Devon K., Dewar K., International Human Genome Sequencing Consortium, Whitehead Institute for Biomedical Research, Center for Genome Research (2001). Initial Sequencing and Analysis of the Human Genome. Nature.

[77] Cremer T., Cremer C. (2001). Chromosome Territories, Nuclear Architecture and Gene Regulation in Mammalian Cells. Nat. Rev. Genet..

[78] Letessier A., Millot G.A., Koundrioukoff S., Lachagès A.-M., Vogt N., Hansen R.S., Malfoy B., Brison O., Debatisse M. (2011). Cell-Type-Specific Replication Initiation Programs Set Fragility of the FRA3B Fragile Site. Nature.

[79] Zeman M.K., Cimprich K.A. (2014). Causes and Consequences of Replication Stress. Nat. Cell Biol..

[80] Durkin S.G., Glover T.W. (2007). Chromosome Fragile Sites. Annu. Rev. Genet..

[81] Weterings E., Chen D.J. (2008). The Endless Tale of Non-Homologous End-Joining. Cell Res..

[82] Mao Z., Bozzella M., Seluanov A., Gorbunova V. (2008). DNA Repair by Nonhomologous End Joining and Homologous Recombination during Cell Cycle in Human Cells. Cell Cycle.

[83] Chiang C., Scott A.J., Davis J.R., Tsang E.K., Li X., Kim Y., Hadzic T., Damani F.N., Ganel L., GTEx Consortium (2017). The Impact of Structural Variation on Human Gene Expression. Nat. Genet..

[84] Liehr T. (2021). Molecular Cytogenetics in the Era of Chromosomics and Cytogenomic Approaches. Front. Genet..

[85] Mitelman F., Johansson B., Mertens F. (2007). The Impact of Translocations and Gene Fusions on Cancer Causation. Nat. Rev. Cancer.

[86] Shaffer L.G., Lupski J.R. (2000). Molecular mechanisms for constitutional chromosomal rearrangements in humans. Annu. Rev. Genet..

[87] Yunis J.J. (1976). High Resolution of Human Chromosomes. Science.

[88] Lichter P., Tang C.-J.C., Call K., Hermanson G., Evans G.A., Housman D., Ward D.C. (1990). High-Resolution Mapping of Human Chromosome 11 by in Situ Hybridization with Cosmid Clones. Science.

[89] Trask B. (1991). Fluorescence in Situ Hybridization: Applications in Cytogenetics and Gene Mapping. Trends Genet..

[90] Lichter P., Cremer T., Borden J., Manuelidis L., Ward D.C. (1988). Delineation of Individual Human Chromosomes in Metaphase and Interphase Cells by in Situ Suppression Hybridization Using Recombinant DNA Libraries. Hum. Genet..

[91] Lawrence J. (1988). Sensitive, High-Resolution Chromatin and Chromosome Mapping in Situ: Presence and Orientation of Two Closely Integrated Copies of EBV in a Lymphoma Line. Cell.

[92] Schröck E., Du Manoir S., Veldman T., Schoell B., Wienberg J., Ferguson-Smith M.A., Ning Y., Ledbetter D.H., Bar-Am I., Soenksen D. (1996). Multicolor Spectral Karyotyping of Human Chromosomes. Science.

[93] Raap A.K., Florijn R.J., Blonden L.A.J., Wiegant J., Vaandrager J.-W., Vrolijk H., Den Dunnen J., Tanke H.J., Van Ommen G.-J. (1996). Fiber FISH as a DNA Mapping Tool. Methods.

[94] Wiegant J., Kalle W., Mullenders L., Brookes S., Hoovers J.M.N., Dauwerse J.G., Van Ommen G.J.B., Raap A.K. (1992). High-Resolution in Situ Hybridization Using DNA Halo Preparations. Hum. Mol. Genet..

[95] Heng H.H., Squire J., Tsui L.C. (1992). High-Resolution Mapping of Mammalian Genes by in Situ Hybridization to Free Chromatin. Proc. Natl. Acad. Sci. USA.

[96] Speicher M.R., Ballard S.G., Ward D.C. (1996). Karyotyping Human Chromosomes by Combinatorial Multi-Fluor FISH. Nat. Genet..

[97] Miller D.T., Adam M.P., Aradhya S., Biesecker L.G., Brothman A.R., Carter N.P., Church D.M., Crolla J.A., Eichler E.E., Epstein C.J. (2010). Consensus Statement: Chromosomal Microarray Is a First-Tier Clinical Diagnostic Test for Individuals with Developmental Disabilities or Congenital Anomalies. Am. J. Hum. Genet..

[98] Coe B.P., Girirajan S., Eichler E.E. (2012). The Genetic Variability and Commonality of Neurodevelopmental Disease. Am. J. Med. Genet. Pt. C.

[99] Gai X., Xie H.M., Perin J.C., Takahashi N., Murphy K., Wenocur A.S., D’arcy M., O’Hara R.J., Goldmuntz E., Grice D.E. (2012). Rare Structural Variation of Synapse and Neurotransmission Genes in Autism. Mol. Psychiatry.

[100] Spielmann M., Mundlos S. (2013). Structural Variations, the Regulatory Landscape of the Genome and Their Alteration in Human Disease. BioEssays.

[101] Mantere T., Kersten S., Hoischen A. (2019). Long-Read Sequencing Emerging in Medical Genetics. Front. Genet..

[102] Mantere T., Neveling K., Pebrel-Richard C., Benoist M., Van Der Zande G., Kater-Baats E., Baatout I., Van Beek R., Yammine T., Oorsprong M. (2021). Optical Genome Mapping Enables Constitutional Chromosomal Aberration Detection. Am. J. Hum. Genet..

[103] Goodwin S., McPherson J.D., McCombie W.R. (2016). Coming of Age: Ten Years of next-Generation Sequencing Technologies. Nat. Rev. Genet..

[104] de Coster W., Weissensteiner M.H., Sedlazeck F.J. (2021). Towards Population-Scale Long-Read Sequencing. Nat. Rev. Genet..

[105] Jain M., Olsen H.E., Paten B., Akeson M. (2016). The Oxford Nanopore MinION: Delivery of Nanopore Sequencing to the Genomics Community. Genome Biol..

[106] Wall B.P.G., Nguyen M., Harrell J.C., Dozmorov M.G., Nakato R. (2025). Machine and deep learning methods for predicting 3D genome organization. Computational Methods for 3D Genome Analysis.

[107] Wenger A.M., Peluso P., Rowell W.J., Chang P.-C., Hall R.J., Concepcion G.T., Ebler J., Fungtammasan A., Kolesnikov A., Olson N.D. (2019). Accurate Circular Consensus Long-Read Sequencing Improves Variant Detection and Assembly of a Human Genome. Nat. Biotechnol..

[108] Oehler J.B., Wright H., Stark Z., Mallett A.J., Schmitz U. (2023). The Application of Long-Read Sequencing in Clinical Settings. Hum. Genom..

[109] Mitelman F., Johansson B., Mertens F. (2022). Mitelman Database of Chromosome Aberrations and Gene Fusions in Cancer. https://mitelmandatabase.isb-cgc.org/.

[110] Druker B.J., Guilhot F., O’Brien S.G., Gathmann I., Kantarjian H., Gattermann N., Deininger M.W.N., Silver R.T., Goldman J.M., Stone R.M. (2006). Five-Year Follow-up of Patients Receiving Imatinib for Chronic Myeloid Leukemia. N. Engl. J. Med..

[111] Bercier P., de Thé H. (2024). History of Developing Acute Promyelocytic Leukemia Treatment and Role of Promyelocytic Leukemia Bodies. Cancers.

[112] Mullighan C.G. (2012). Molecular Genetics of B-Precursor Acute Lymphoblastic Leukemia. J. Clin. Investig..

[113] Melo J.V., Barnes D.J. (2007). Chronic Myeloid Leukaemia as a Model of Disease Evolution in Human Cancer. Nat. Rev. Cancer.

[114] Rowley J.D. (1973). A New Consistent Chromosomal Abnormality in Chronic Myelogenous Leukaemia Identified by Quinacrine Fluorescence and Giemsa Staining. Nature.

[115] Garimberti E., Federico C., Ragusa D., Bruno F., Saccone S., Bridger J.M., Tosi S. (2024). Alterations in Genome Organization in Lymphoma Cell Nuclei Due to the Presence of the t(14;18) Translocation. Int. J. Mol. Sci..

[116] Tsujimoto Y., Finger L.R., Yunis J., Nowell P.C., Croce C.M. (1984). Cloning of the Chromosome Breakpoint of Neoplastic B Cells with the t(14;18) Chromosome Translocation. Science.

[117] Dang C.V. (2012). MYC on the Path to Cancer. Cell.

[118] Hnisz D., Weintraub A.S., Day D.S., Valton A.-L., Bak R.O., Li C.H., Goldmann J., Lajoie B.R., Fan Z.P., Sigova A.A. (2016). Activation of Proto-Oncogenes by Disruption of Chromosome Neighborhoods. Science.

[119] Ebert B.L., Pretz J., Bosco J., Chang C.Y., Tamayo P., Galili N., Raza A., Root D.E., Attar E., Ellis S.R. (2008). Identification of RPS14 as a 5q- Syndrome Gene by RNA Interference Screen. Nature.

[120] Lv L., Yu J., Qi Z. (2020). Acute Myeloid Leukemia with Inv(16)(P13.1q22) and Deletion of the 5’MYH11/3’CBFB Gene Fusion: A Report of Two Cases and Literature Review. Mol. Cytogenet..

[121] Olivier M., Hollstein M., Hainaut P. (2010). TP53 Mutations in Human Cancers: Origins, Consequences, and Clinical Use. Cold Spring Harb. Perspect. Biol..

[122] The Cancer Genome Atlas Network (2015). Comprehensive Genomic Characterization of Head and Neck Squamous Cell Carcinomas. Nature.

[123] Cairncross J.G., Wang M., Jenkins R.B., Shaw E.G., Giannini C., Brachman D.G., Buckner J.C., Fink K.L., Souhami L., Laperriere N.J. (2014). Benefit From Procarbazine, Lomustine, and Vincristine in Oligodendroglial Tumors Is Associated with Mutation of *IDH*. J. Clin. Oncol..

[124] Korbel J.O., Campbell P.J. (2013). Criteria for Inference of Chromothripsis in Cancer Genomes. Cell.

[125] Beroukhim R., Mermel C.H., Porter D., Wei G., Raychaudhuri S., Donovan J., Barretina J., Boehm J.S., Dobson J., Urashima M. (2010). The Landscape of Somatic Copy-Number Alteration across Human Cancers. Nature.

[126] Aaltonen L.A., Abascal F., Abeshouse A., Aburatani H., Adams D.J., Agrawal N., Ahn K.S., Ahn S.-M., Aikata H., The ICGC/TCGAPan-Cancer Analysis of Whole Genomes Consortium (2020). Pan-Cancer Analysis of Whole Genomes. Nature.

[127] Rowley M.J., Corces V.G. (2018). Organizational Principles of 3D Genome Architecture. Nat. Rev. Genet..

[128] Dekker J., Mirny L. (2016). The 3D Genome as Moderator of Chromosomal Communication. Cell.

[129] Dryden N.H., Broome L.R., Dudbridge F., Johnson N., Orr N., Schoenfelder S., Nagano T., Andrews S., Wingett S., Kozarewa I. (2014). Unbiased Analysis of Potential Targets of Breast Cancer Susceptibility Loci by Capture Hi-C. Genome Res..

[130] The ENCODE Project Consortium (2012). An Integrated Encyclopedia of DNA Elements in the Human Genome. Nature.

[131] Barski A., Cuddapah S., Cui K., Roh T.-Y., Schones D.E., Wang Z., Wei G., Chepelev I., Zhao K. (2007). High-Resolution Profiling of Histone Methylations in the Human Genome. Cell.

[132] Buenrostro J.D., Giresi P.G., Zaba L.C., Chang H.Y., Greenleaf W.J. (2013). Transposition of Native Chromatin for Fast and Sensitive Epigenomic Profiling of Open Chromatin, DNA-Binding Proteins and Nucleosome Position. Nat. Methods.

[133] Emerson D.J., Zhao P.A., Cook A.L., Barnett R.J., Klein K.N., Saulebekova D., Ge C., Zhou L., Simandi Z., Minsk M.K. (2022). Cohesin-Mediated Loop Anchors Confine the Locations of Human Replication Origins. Nature.

[134] Franke M., Ibrahim D.M., Andrey G., Schwarzer W., Heinrich V., Schöpflin R., Kraft K., Kempfer R., Jerković I., Chan W.-L. (2016). Formation of New Chromatin Domains Determines Pathogenicity of Genomic Duplications. Nature.

[135] Lupiáñez D.G., Kraft K., Heinrich V., Krawitz P., Brancati F., Klopocki E., Horn D., Kayserili H., Opitz J.M., Laxova R. (2015). Disruptions of Topological Chromatin Domains Cause Pathogenic Rewiring of Gene-Enhancer Interactions. Cell.

[136] Dekker J., Belmont A.S., Guttman M., Leshyk V.O., Lis J.T., Lomvardas S., Mirny L.A., O’Shea C.C., Park P.J., The 4D Nucleome Network (2017). The 4D Nucleome Project. Nature.

[137] Dixon J.R., Jung I., Selvaraj S., Shen Y., Antosiewicz-Bourget J.E., Lee A.Y., Ye Z., Kim A., Rajagopal N., Xie W. (2015). Chromatin Architecture Reorganization during Stem Cell Differentiation. Nature.

[138] Liu N., Li X., Luo X., Liu B., Tang J., Xiao F., Wang W., Tang Y., Shu P., Zhang B. (2025). Development and Validation of Machine Learning Models Based on Molecular Features for Estimating the Probability of Multiple Primary Lung Carcinoma versus Intrapulmonary Metastasis in Patients Presenting Multiple Non-Small Cell Lung Cancers. Transl. Lung Cancer Res..

[139] Pang A.W., MacDonald J.R., Pinto D., Wei J., Rafiq M.A., Conrad D.F., Park H., Hurles M.E., Lee C., Venter J.C. (2010). Towards a Comprehensive Structural Variation Map of an Individual Human Genome. Genome Biol..

[140] Duan G., Huo Q., Ni W., Ding F., Ye Y., Tang T., Dai H. (2025). Integrative Machine Learning Model for Subtype Identification and Prognostic Prediction in Lung Squamous Cell Carcinoma. Discov. Onc.

[141] Lareau C.A., Duarte F.M., Chew J.G., Kartha V.K., Burkett Z.D., Kohlway A.S., Pokholok D., Aryee M.J., Steemers F.J., Lebofsky R. (2019). Droplet-Based Combinatorial Indexing for Massive-Scale Single-Cell Chromatin Accessibility. Nat. Biotechnol..

[142] Nagano T., Lubling Y., Várnai C., Dudley C., Leung W., Baran Y., Mendelson Cohen N., Wingett S., Fraser P., Tanay A. (2017). Cell-Cycle Dynamics of Chromosomal Organization at Single-Cell Resolution. Nature.

[143] Dekker J., Marti-Renom M.A., Mirny L.A. (2013). Exploring the Three-Dimensional Organization of Genomes: Interpreting Chromatin Interaction Data. Nat. Rev. Genet..

[144] Shendure J., Balasubramanian S., Church G.M., Gilbert W., Rogers J., Schloss J.A., Waterston R.H. (2017). DNA Sequencing at 40: Past, Present and Future. Nature.

[145] Buenrostro J.D., Wu B., Chang H.Y., Greenleaf W.J. (2015). ATAC-seq: A Method for Assaying Chromatin Accessibility Genome-Wide. Curr. Protoc. Mol. Biol..

[146] Shao Y., Lv X., Ying S., Guo Q. (2024). Artificial Intelligence-Driven Precision Medicine: Multi-Omics and Spatial Multi-Omics Approaches in Diffuse Large B-Cell Lymphoma (DLBCL). Front. Biosci..

[147] Zhang Y., Boninsegna L., Yang M., Misteli T., Alber F., Ma J. (2024). Computational Methods for Analysing Multiscale 3D Genome Organization. Nat. Rev. Genet..

[148] Van Karnebeek C.D.M., Wortmann S.B., Tarailo-Graovac M., Langeveld M., Ferreira C.R., Van De Kamp J.M., Hollak C.E., Wasserman W.W., Waterham H.R., Wevers R.A. (2018). The Role of the Clinician in the Multi-omics Era: Are You Ready?. J. Inher Metab. Disea.

[149] O’Connor O., McVeigh T.P. (2025). Increasing Use of Artificial Intelligence in Genomic Medicine for Cancer Care- the Promise and Potential Pitfalls. BJC Rep..

[150] Lin M., Guo J., Gu Z., Tang W., Tao H., You S., Jia D., Sun Y., Jia P. (2025). Machine Learning and Multi-Omics Integration: Advancing Cardiovascular Translational Research and Clinical Practice. J. Transl. Med..

